# Impaired activation of lesional CD8^+^ T-cells is associated with enhanced expression of Programmed Death-1 in Indian Post Kala-azar Dermal Leishmaniasis

**DOI:** 10.1038/s41598-018-37144-y

**Published:** 2019-01-24

**Authors:** Shibabrata Mukherjee, Ritika Sengupta, Debanjan Mukhopadhyay, Claudia Braun, Sneha Mitra, Susmita Roy, Nilay Kanti Das, Uttara Chatterjee, Esther von Stebut, Mitali Chatterjee

**Affiliations:** 1 Department of Pharmacology, Institute of Postgraduate Medical Education and Research, Kolkata, 700020 India; 2 Department of Dermatology, University Medical Center, Johannes Gutenberg University, Mainz, 55131 Germany; 3 Department of Dermatology, Calcutta Medical College, Kolkata, 700073 India; 4 Department of Pathology, Institute of Postgraduate Medical Education and Research, Kolkata, 700020 India; 5 Klinik für Dermatologie und Venerologie, Universitätsklinikum Köln, 50937 Koln, Germany; 6 Present Address: Department of Pathology, Microbiology and Immunology, School of Veterinary Medicine, University of California, Davis, USA

**Keywords:** Chemokines, Interleukins, Immune evasion, CD8-positive T cells, Parasitic infection

## Abstract

Post Kala-azar dermal leishmaniasis (PKDL), caused by *Leishmania donovani* is the dermal sequel of Visceral Leishmaniasis and importantly, is the proposed disease reservoir. The survival of *Leishmania* parasites within monocytes/macrophages hinges on its ability to effectively nullify immune activation mechanisms. Thus, delineating the disease-promoting immune mechanisms can facilitate development of immunotherapeutic strategies. Accordingly, in the absence of an animal model, this study aimed to delineate the status of CD8^+^ T-cells in patients with PKDL. At disease presentation, the absence of CD4^+^ T-cells at lesional sites was concomitant with an overwhelming infiltration of CD8^+^ T-cells that demonstrated an absence of Perforin, Granzyme and Zap-70, along with an enhanced expression of Programmed Death-1 (PD-1) and the skin-homing CCL17. Additionally, the lesional CCR4^+^CD8^+^ population was associated with an enhanced expression of IL-10 and IL-5. In circulation, the enhanced CD8^+^CCR4^+^ T-cell population and raised levels of CCL17/22 was associated with an increased frequency of PD-1, while CD127 was decreased. Taken together, in PKDL, the enhanced plasma and lesional CCL17 accounted for the dermal homing of CD8^+^CCR4^+^ T-cells, that along with a concomitant upregulation of PD-1 and IL-10 mediated immune inactivation, emphasizing the need for designing immunotherapies capable of reinvigorating T-cell potency.

## Introduction

The Leishmaniases, caused by the protozoan parasite *Leishmania*, comprise a diverse group of neglected tropical diseases having a range of clinical features varying from innocuous self-healing cutaneous lesions to fatal visceralization or a metastatic dermal dissemination. Post Kala-azar Dermal Leishmaniasis (PKDL), usually presents in patients with a history of treated Visceral Leishmaniasis (VL) caused by *L*. *donovani* and is possibly the most challenging variant of Leishmaniasis, especially in terms of its etiopathogenesis^1,2^. Patients with PKDL present with papulonodular (polymorphic) or hypomelanotic lesions (macular), and the disease is confined to South Asia and East Africa (mainly Sudan). In South Asia, approximately 5–10% of apparently cured VL patients develop PKDL, as against 50–60% in Sudan^3,4^. As VL is anthroponotic, PKDL cases are considered as the disease reservoir, emphasizing their inclusion as a component of the ongoing VL elimination programme^5,6^. In order to achieve this goal of elimination, it is important to delineate the pathophysiology so that informed decisions can be made regarding the most appropriate and cost effective treatment approach^7^. This necessitates an understanding of the parasite-driven immune evasion strategies evolved in PKDL that enable parasite survival following apparent cure from VL^8,9^.

Intracellular pathogens like *Leishmania* have evolved innovative approaches to evade immune responses that include interference with antigen processing/presentation, altered phagocytosis, induction of immune regulatory pathways and manipulation of costimulatory molecules^10^. Accordingly, the outcome of *Leishmania* infections is influenced by functionally distinct T-cell populations, namely Th1 (IL-2, IFN-γ, IL-6, TNF-α etc.), Th2 (IL-4, IL-13) and Tr1 (IL-10, TGF-β)^11^. Cutaneous Leishmaniasis (CL), is possibly the best documented example of differential activation, wherein disease susceptibility is associated with a predominant Th2 proliferation, while healing responses are associated with an expansion of IFN-γ producing CD4^+^ Th1 cells, secondary to production of IL-12^11^. In VL, the disease is less defined and is associated with a mixed Th1/Th2 immune profile, along with impairment of macrophage functions^12–14^. Akin to VL, the pathobiology of PKDL involves an enhanced Th1/Th2 response with a Th2 bias, as evident by increased levels of IL-4, IL-5, IL-13, IL-10 and TGF-β, with a preponderance of circulating CD8^+^IL-10^+^ T-cells^15–19^.

In PKDL, a disease where no animal model exists, information is derived solely from human studies, and understandably remains limited. Studies have endorsed the presence of a systemic and dermal immunosuppressive milieu and includes the presence of an increased population of antigen-specific IL-10 producing anergic T-cell population in peripheral blood^20^, a decreased presence of dendritic cells at lesional sites^21^, dampening of the CD26 regulated pathways^22^, a huge infiltration of CD68^+^ alternatively activated macrophages^23^ and a dermal pathology dominated by IL-10 and FoxP3^15,17,20^, that individually or more likely collectively contribute towards establishment of a pro-parasitic milieu. In the peripheral blood of polymorphic PKDL as compared to the macular variant, stimulation with *Leishmania* antigen enhanced levels of activated CD8^+^ and CD4^+^ T cells^24^. However, what remains poorly defined in PKDL is the status of chemokines and T-cells at the lesional sites, along with defining their contribution, if any, in supporting disease progression. Accordingly in this study, the activation status of CD4^+^ and CD8^+^ T-cells, cytotoxic markers e.g. Perforin, Granzyme and p-Zap-70, inhibitory receptor- Programmed death-1 (PD-1), skin homing chemokine CCL17 and its receptor, Chemokine Receptor 4 (CCR4) along with IL-5 and IL-10 were evaluated in dermal lesions of patients with PKDL. The results demonstrated an increased proportion of CD8^+^CCR4^+^ T-cells and CCL17/CCL22 indicative of dermal homing, while the upregulation of PD-1 and IL-10 suggested impaired activation of CD8^+^ T-cells. Taken together, this dermal homing of anergic/exhausted CD8^+^ T-cells supported parasite survival and disease progression in patients with PKDL.

## Results

The study population included patients with PKDL (n = 20) recruited from 2004–2014, whose median age was 27.50 years with a male preponderance (Table 1)^3,25,26^. The majority demonstrated hypopigmented, papular and/or nodular lesions, termed ‘polymorphic’ (n = 18, 90.0%), while a minority presented with hypopigmented lesions termed ‘macular’ (n = 2, 10.0%). The papular/nodular lesions appeared primarily on sun-exposed areas like face, neck and upper limbs and ranged from 10–12 in number, whereas for the macular variant, the distribution was more diffuse (Supplementary Fig. S1). All were ITS-1 PCR positive whereas Leishman-Donovan (LD) bodies were detected only in Giemsa stained smears of the polymorphic variant. Two patients gave no prior history of VL, while in the remaining 18, the mean time interval between cure from VL and onset of PKDL was 2.75 years (Table 1). Following completion of treatment, polymorphic PKDL cases showed complete resolution of papules/nodules whereas in macular PKDL, there was substantial reduction in hypopigmentation (Supplementary Fig. S1).

**Table 1:** Study population.

Characteristics	Patients with PKDL (n = 20)	Healthy controls (n = 10)
Age (years)^*^	27.50 (18.25–34.35)	30.43 (19.56–37.45)
Sex ratio (M:F)	3.4:1	4:1
Disease duration (years)^*^	2.71 (1.00–11.20)	NA
Interval between cure of VL and onset of PKDL in years^*^	2.75 (1.00–23.00)	NA

^*^Values are expressed as median (IQR).

M = Male; F = Female; NA = Not applicable; VL = Visceral Leishmaniasis.

### Extensive dermal infiltration and absence of granuloma are features of Indian PKDL

An overall histopathological analysis by H&E staining showed a dense, diffuse inflammatory cell infiltrate involving the entire dermis, consisting mainly of lymphocytes, macrophages and plasma cells, and varied from severe in the polymorphic cases to patchy perivascular and periappendageal with relative sparing of the reticular dermis in the macular variant. Other features included an atrophic epidermis, follicular plugging, hyperkeratosis and papillomatosis. Unlike leprosy, the narrow sub-epidermal Grenz zone was spared and there were no well-formed granuloma. The neural Schwann cells remained unaffected and did not harbour parasites; furthermore, except for one case where perineural infiltration was prominent^27^, none reported any loss of sensation or involvement of the sub-cutis (Supplementary Fig. S2).

### CD8^+^ T-cells predominated in the dermal infiltrate

**Figure 1:**
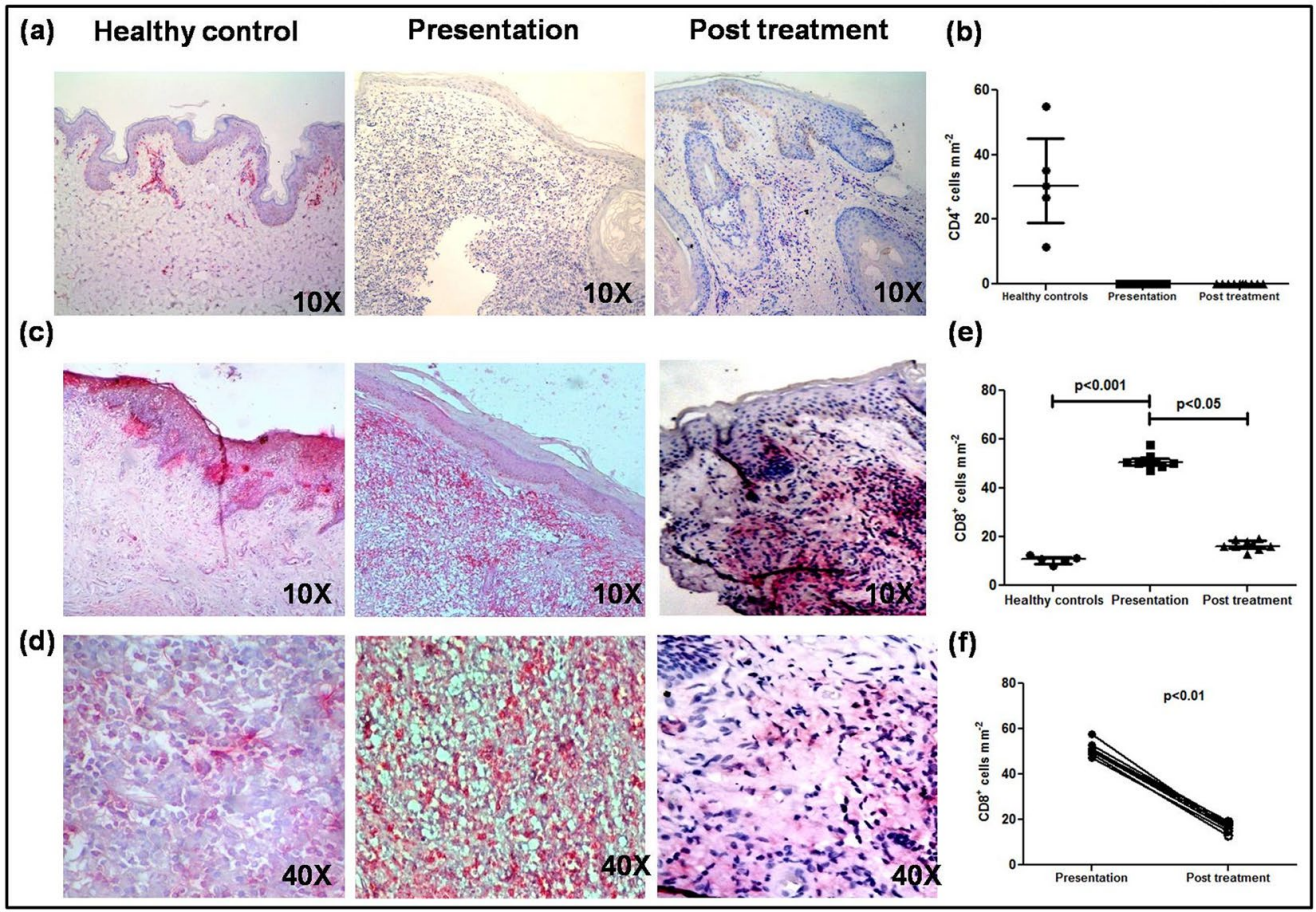
Distribution of T-cells in dermal lesions of patients with PKDL. (**a**) Representative immunohistochemical profiles of CD4^+^ T-cells from dermal biopsies of a healthy control, patient with PKDL and post treatment (10X magnification). (**b**) Scatter plots of CD4^+^ T-cells in dermal biopsies of healthy controls (●, n = 5) patients with PKDL (■, n = 10) and post treatment (▲, n = 10). Each horizontal bar represents the median value. (**c**,**d**) Representative immunohistochemical profiles of CD8^+^ T-cells from dermal biopsies of a healthy control, patient with PKDL and post treatment (10X and 40X magnification). (**e**) Scatter plots of CD8^+^ T-cells in dermal biopsies of healthy controls (●, n = 5) patients with PKDL (■, n = 9) and post treatment (▲, n = 9). Each horizontal bar represents the median value. (**f**) Before (●) and after (○) treatment plots of CD8^+^ T-cells.

In Indian PKDL Rathi *et al*.^28^ reported a preponderance of T-suppressor over T-helper cells but as the cellular phenotype had not been characterized, further studies are warranted, especially to establish their activation status. Immunohistochemical analysis at the lesional site showed a conspicuous absence of CD4^+^ T-cells, *vis-a-vis* healthy controls [0 vs. 30.45 (19.00–45.13), Fig. 1a,b] that remained so even after treatment (Fig. 1a,b). CD4^+^ staining was confirmed in human lymph node sections (Supplementary Fig. S3). However, the proportion of CD8^+^ T-cells was 4.61 fold higher than controls, 50.30(49.30–52.05) vs. 10.9(9.04–11.81), p < 0.001 (Fig. 1c–e). Importantly, there was a strong correlation between the frequency of CD8^+^ T-cells and disease duration, r = 0.70 (CI = −0.15–0.94). With treatment, a dramatic decline occurred, 16.21(15.37–18.33), p < 0.05 (Fig. 1c–e), which was evident even on an individual basis, p < 0.01 (Fig. 1f). Flow cytometric analysis of peripheral blood showed that the proportion of CD4^+^ and CD8^+^ T-cells remained unchanged and was comparable with healthy controls.

### Status of CCL17 and CCL22 in PKDL

**Figure 2:**
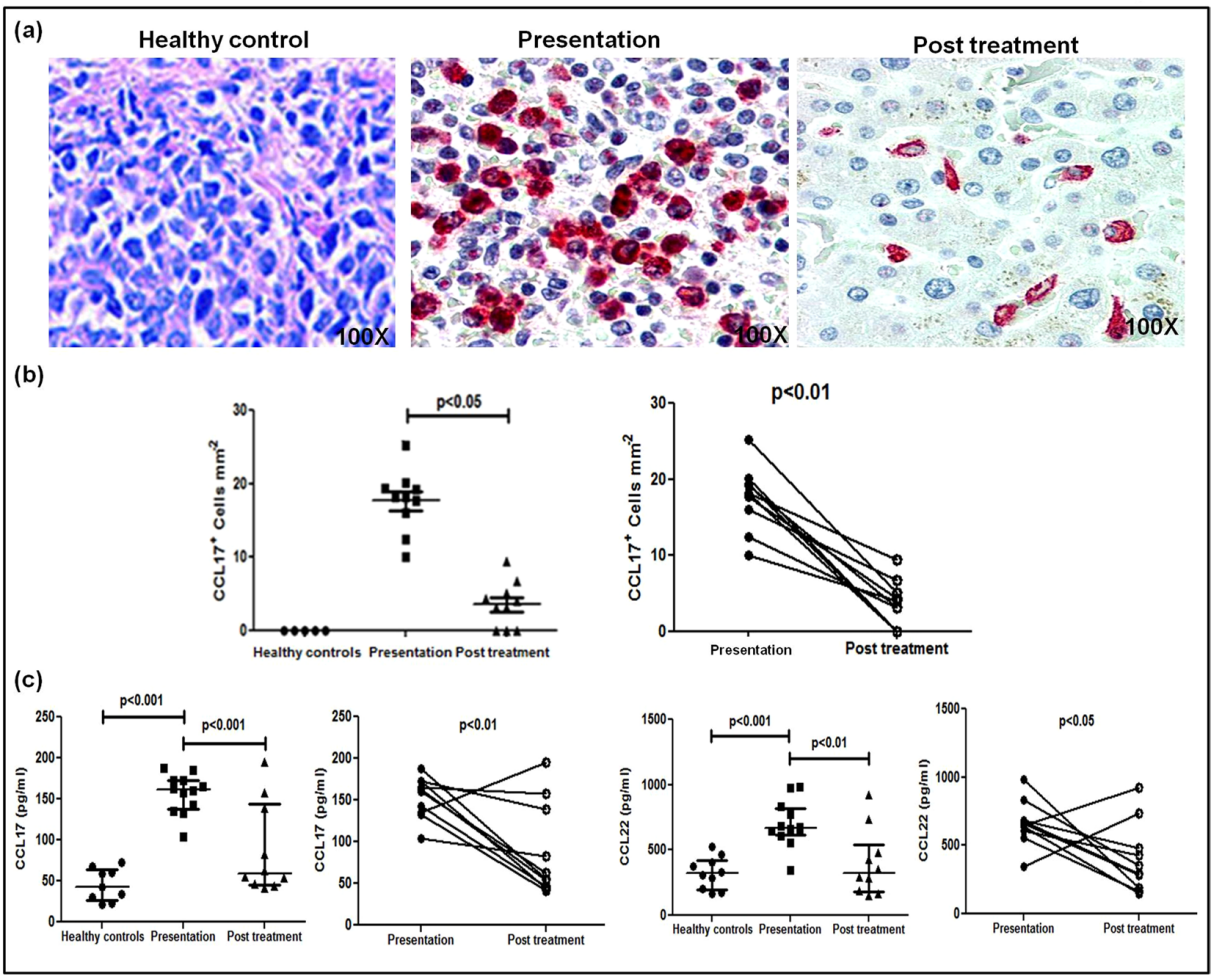
Dermal homing of CD8^+^ T-cells in patients with PKDL. (**a**) Representative immunohistochemical profiles of CCL17^+^ cells from dermal biopsies of a healthy control, patient with PKDL and post treatment (100X magnification). (**b**) Scatter plots showing the status of dermal CCL17^+^ cells in healthy controls (●, n = 5), patients with PKDL at presentation (■, n = 10), and post treatment (▲, n = 10), along with before (●) and after (○) treatment plots. Each horizontal bar represents the median value. (**c**) Scatter plots showing plasma levels of CCL17 and CCL22 in healthy controls (●, n = 9 for CCL17, n = 10 for CCL22), patients with PKDL (■, n = 12), and post treatment (▲, n = 10); each horizontal bar represents the median value. Before (●) and after (○) treatment plots of CCL17 and CCL22.

In PKDL, there is an increased presence of alternatively activated CD68^+^ macrophages^21,23^ that possibly supported an increased presence of T-cell chemokines CCL17/CCL22, accounting for the increased presence of CD8^+^ T-cells^29^. Immunohistochemical analysis of the lesional site showed a significantly higher proportion of CCL17^+^ cells, mainly histiocytes, *vis-a-vis* their absence in healthy controls, 18.19(15.13–19.58) vs. 0 (Fig. 2a,b). Treatment translated into a significant decrease, being 4.21(2.13–8.24), p < 0.05, and was corroborated on an individual basis, p < 0.01 (Fig. 2a,b).

The circulating levels of CCL17 as measured using a multiplex ELISA based assay were significantly up regulated as compared to healthy controls, 161.60(137.40–173.20) vs. 43.20(26.36–64.50) pg/ml, p < 0.001, and decreased with treatment, 58.69(45.50–144.10) pg/ml, p < 0.001 (Fig. 2c). Similarly, levels of CCL22 were also significantly raised, 665.90(614.40–819.20) vs. 321.10(195.30–423.10) pg/ml, p < 0.001, and declined significantly with treatment to 323.20(181.40–544.00) pg/ml, p < 0.01 (Fig. 2c). However, in one case post treatment, levels of CCL17 increased and in three cases remained unchanged. With regard to CCL22, levels were increased post treatment in two patients. Overall on a paired basis, both these chemokines demonstrated a significant decrease (Fig. 2c).

### Increased CD8^+^CCR4^+^ cells in dermal lesions and peripheral blood

**Figure 3:**
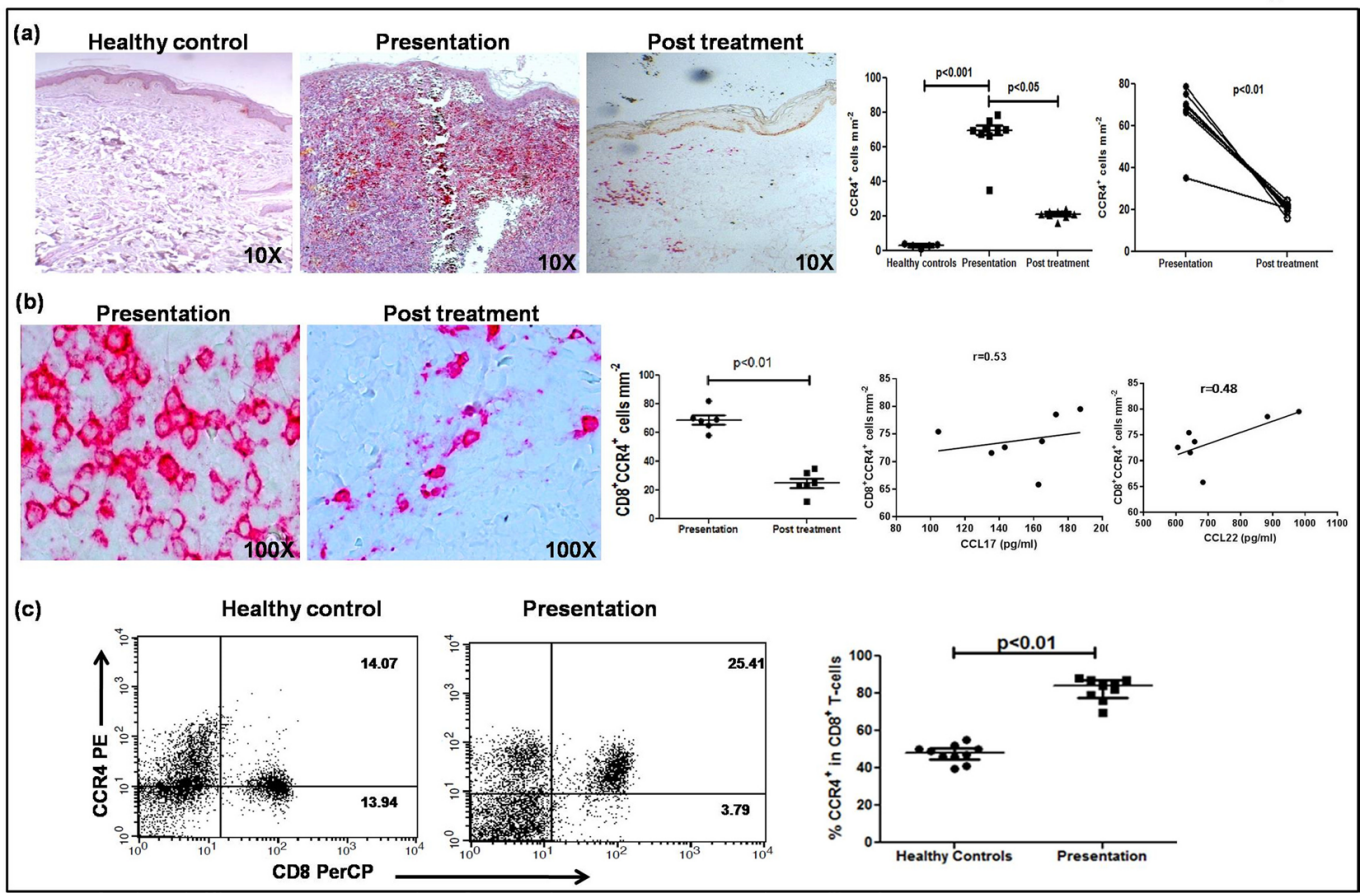
Status of Chemokine Receptor 4 (CCR4) in CD8^+^ T-cells in patients with PKDL. (**a**) Representative immunohistochemical profiles of CCR4^+^ T-cells from dermal biopsies of a healthy control, patient with PKDL and post treatment (10X magnification). Scatter plots showing the status of dermal CCR4^+^ cells in healthy controls (●, n = 5), patients with PKDL (■, n = 9), and post treatment (▲, n = 9), along with before (●) and after (○) treatment plots. Each horizontal bar represents the median value. (**b**) Representative immunohistochemical profiles of CD8^+^CCR4^+^ T-cells from dermal biopsies of a patient with PKDL and post treatment (100X magnification); in the co-localization stain, red denotes the CD8 staining and brown denotes the CCR4 stain, along with scatter plots of CD8^+^CCR4^+^T-cells at disease presentation (●, n = 6) and post treatment (■, n = 6). Each horizontal bar represents the median value. Correlation of CD8^+^CCR4^+^ T-cells with plasma levels of CCL 17 and CCL 22 respectively. (**c**) Representative quadrant plots of CD8^+^CCR4^+^ T-cells in peripheral blood of a healthy control and a patient with PKDL. Scatter plot of CCR4^+^ cells in CD8^+^ T-cells of healthy controls (●, n = 10) and at disease presentation (■, n = 10). The proportion of CCR4^+^ cells in the entire CD8^+^ population was calculated by dividing the percentages of upper right quadrant by the sum of the upper and lower right quadrant. Each horizontal bar represents the median value.

The raised levels of dermal homing chemokines CCL17 and CCL22 (Fig. 2a–c) suggested that the increased presence of CD8^+^ T-cells was secondary to their homing to dermal lesions^30^. Accordingly, the status of CCR4, the chemokine receptor for CCL17/22 was examined at dermal sites by immunohistochemistry and flow cytometry in peripheral blood. In dermal lesions, the proportion of CCR4^+^ cells was significantly up regulated, 69.56(67.09–72.78) vs. 3.26(2.11–3.88), p < 0.001 (Fig. 3a) and declined significantly with treatment, 21.23(19.96–22.35), p < 0.05, and was confirmed on an individual basis, p < 0.01 (Fig. 3a). Dual staining confirmed the localization of CCR4 on CD8^+^ T-cells (Fig. 3b), which decreased with treatment, 65.85(43.56–73.65) vs. 20.20(8.52–15.13), p < 0.01 (Fig. 3b). Furthermore, the raised plasma levels of CCL17 and 22 correlated positively with the proportion of dermal CD8^+^/CCR4^+^ T-cells, r = 0.53 and r = 0.48 respectively (Fig. 3b). In peripheral blood, the frequency of CCR4^+^ within the CD8^+^ population was significantly higher than healthy controls, 81.98(64.71–34.23) vs. 49.87(39.85–58.36)%, p < 0.01 (Fig. 3c) whereas the frequency of CXCR3^+^ within the CD8^+^ population remained unaltered, and was comparable with healthy controls, 27.20 (25.90–31.25) vs. 27.10 (23.85–30.80)% (Supplementary Fig. S4).

### Decreased expression of granzyme and perforin in lesional CD8^+^ T-cells

**Figure 4:**
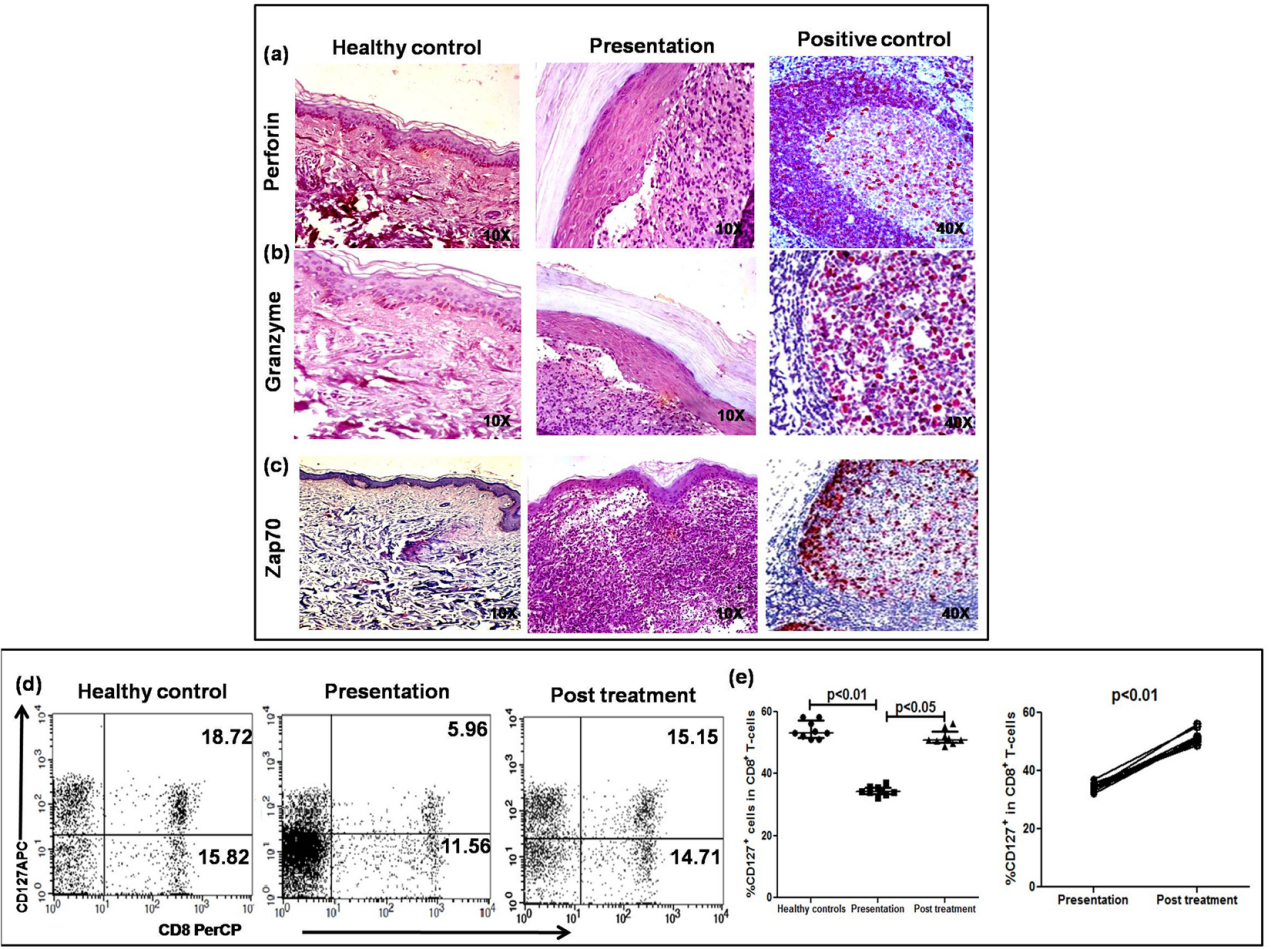
Activation status of lesional and peripheral CD8^+^ T-cells in patients with PKDL. (**a–c**) Representative immunohistochemical profiles of Perforin (**a**), Granzyme (**b**) and Zap70 (**c**) in dermal biopsies of a healthy control, patient with PKDL (10X magnification); a reactive lymph node served as the positive control (40X magnification). (**d**) Representative quadrant plots indicating frequency (%) of CD127^+^ within CD8^+^ T-cells in peripheral blood of a healthy control, patient with PKDL and post treatment. (**e**) Scatter plots showing frequency (%) of CD127^+^ within CD8^+^ T-cells of healthy controls (●, n = 9), patients with PKDL (■, n = 10) and post treatment (▲, n = 9). The proportion of CD127^+^ within the entire CD8^+^ population was calculated by dividing the percentages of upper right quadrant by the sum of upper and lower right quadrant. Each horizontal bar represents the median value. Before (●) and after (○) treatment plots of CD127^+^ in CD8^+^ T-cells in patients with PKDL.

To assess the cytotoxic potential of lesional CD8^+^ T-cells, the lesional status of Perforin-H and Granzyme-B along with nuclear factor Zap70 was examined by immunohistochemistry^31^. Despite an overwhelming presence of CD8^+^ T-cells, PKDL cases demonstrated a conspicuous absence of Perforin, Granzyme and Zap70 in the dermal lesions (Fig. 4a–c). In peripheral blood, the activation status of CD8^+^ T-cells was examined by flow cytometry in terms of activation markers, CD127 and CD69^32,33^ along with Perforin and Granzyme. During active disease, the frequency of CD127 within CD8^+^ T-cells was significantly decreased, 36.14(33.60–38.52) vs. 55.26(51.70–59.15) %, p < 0.01 (Fig. 4d,e) and with treatment, increased significantly to 51.87(50.18–55.69) %, p < 0.05 (Fig. 4d,e), and was evident on an individual basis, p < 0.01. However, the frequency of CD69, Granzyme and Perforin in circulating CD8^+^ T-cells remained unchanged and was comparable with healthy controls being 8.36(6.43–10.03) vs. 8.01(6.65–12.64) % for CD69, 22.22(19.89–25.87) vs. 23.44(19.18–25.33) % for Granzyme and 19.19(17.80–23.29) vs. 18.96 (13.57–20.03) % for Perforin (Supplementary Fig. S5).

### Enhanced presence of markers of exhaustion in lesional and circulatory CD8^+^ T-cells

**Figure 5:**
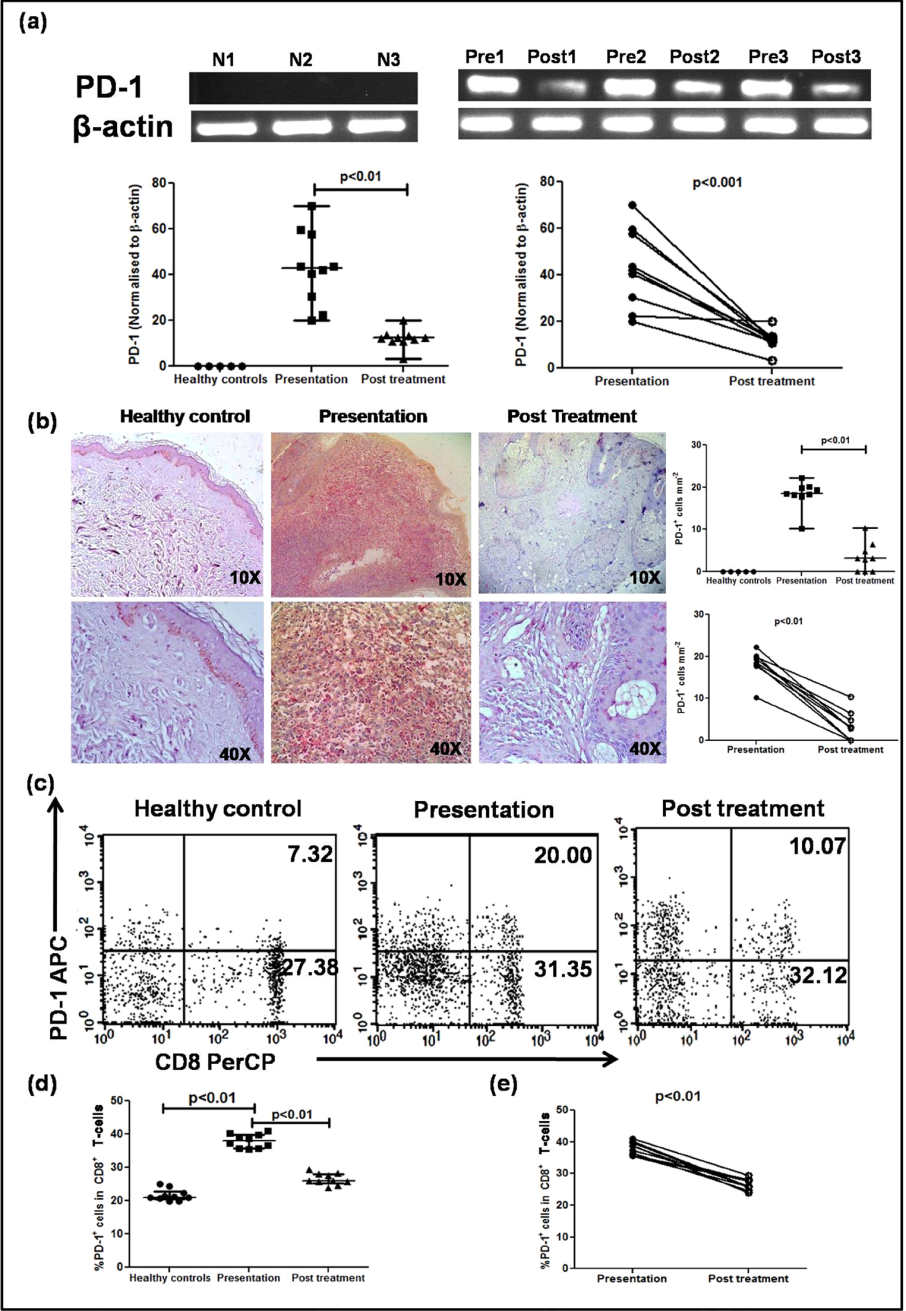
Presence of Programmed Death-1 (PD-1) in dermal lesions and circulation of patients with PKDL. (**a**) Representative mRNA expression profiles of PD-1 (cropped from original gel images, full-length gels are presented in Supplementary Fig. S6) in dermal biopsies of healthy controls (N1–3), patients with PKDL (Pre1–3) and post treatment (Post1–3). Scatter plots of PD-1 in healthy controls (●, n = 5), at disease presentation (■, n = 10) and post treatment (▲, n = 10). Each horizontal bar represents the median value. Before (●) and after (○) treatment plots of PD-1. (**b**) Representative immunohistochemical profiles of PD-1^+^ cells from dermal biopsies of a healthy control, patient with PKDL and post treatment (10X and 40X magnification). Scatter plots of PD-1^+^ cells in dermal biopsies of healthy controls (●, n = 5) patients with PKDL (■, n = 9) and post treatment (▲, n = 9). Each horizontal bar represents the median value. Before (●) and after treatment (○) plots of PD-1^+^ cells. (**c**) Representative quadrant plots indicating the frequency (%) of PD-1^+^ within CD8^+^ T-cells in peripheral blood of a healthy control, patient with PKDL and post treatment. (**d**) Scatter plots of PD-1^+^ in CD8^+^ T-cells of healthy controls (●, n = 10), at disease presentation (■, n = 10) and post treatment (▲, n = 10). Each horizontal bar represents the median value. The proportion of PD-1^+^ in the entire CD8^+^ population was calculated by dividing percentages of the upper right quadrant by the sum of upper and lower right quadrant. (**e**) Before (●) and after treatment (○) plots of PD-1^+^ in CD8^+^ T-cells.

The absence of cytotoxic markers Perforin, Granzyme, Zap-70 in dermal lesions suggested exhaustion of CD8^+^ T-cells and accordingly, the presence of Programme Death 1 (PD-1), a marker of exhaustion was examined^34,35^. In dermal lesions, the mRNA expression of PD-1 was elevated, 42.86(28.42–58.28) vs. 0 (Fig. 5a), and regressed significantly with treatment, 12.35(10.93–13.49), p < 0.01 (Fig. 5a). This was corroborated on an individual basis, p < 0.001 (Fig. 5a). Furthermore, a significant increase in PD-1 expression was also demonstrated by immunohistochemistry in dermal lesions, absent in healthy individuals, 18.50(17.94–20.03) vs. 0 (Fig. 5b); treatment translated into a significant decrease, 3.14(0–5.68), p < 0.01 (Fig. 5b). The scenario was mirrored in peripheral blood as PKDL cases demonstrated a significantly elevated frequency of PD-1 within CD8^+^ T-cells, 38.03(35.75–39.84) vs. 21.03(20.45–22.84) %, p < 0.01 (Fig. 5c,d), which decreased with treatment, 24.88(20.37–28.00) %, p < 0.01(Fig. 5c,d), and this trend was evident even on an individual basis, p < 0.01 (Fig. 5e).

**Figure 6:**
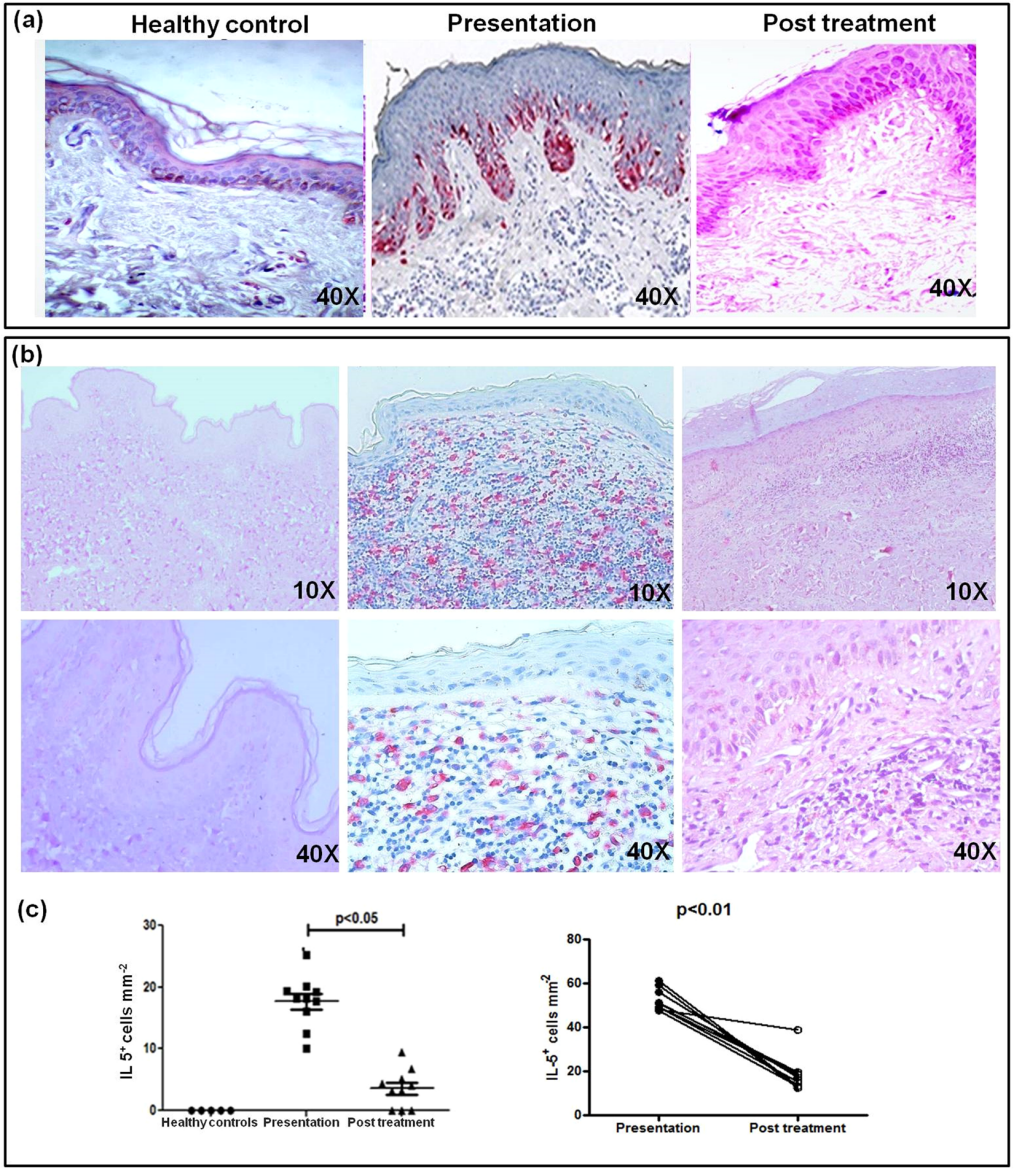
Distribution of IL-10 and IL-5 in dermal lesions of patients with PKDL. (**a**) Representative immunohistochemical profiles of IL-10 from dermal biopsies of a healthy control, a patient with PKDL and post treatment (40X magnification). (**b**) Representative immunohistochemical profiles of IL-5 from dermal biopsies of a healthy control, a patient with PKDL and post treatment (10X and 40X magnification). (**c**) Scatter plots showing distribution of IL-5^+^ cells in healthy controls (●, n = 5), at disease presentation (■, n = 10) and post treatment (▲, n = 10). Each horizontal bar represents the median value. Before (●) and after (○) treatment plots of IL-5^+^ cells.

As exhaustion is associated with an immunosuppressive milieu comprising IL-4 and IL-10^35^, their plasma levels in PKDL were examined and found to be significantly raised, and with treatment a significant decrease was reported^16^. Furthermore, as the sustenance of CCR4^+^ T-cells (Fig. 3) is generally supported by an IL-10 and IL-5 rich milieu^36,37^, their expression was examined at lesional sites. During active disease, there was a strongly positive diffuse sub-epidermal expression of IL-10 which receded with treatment (n = 10, Fig. 6a). Concomitantly, an enhanced expression of IL-5 was evident, 18.12(41.35–60.14) vs. 0 (Fig. 6b,c), which also declined with treatment, 4.30(10.21–31.25), p < 0.05 (Fig. 6b,c); this was mirrored on an individual basis, p < 0.01 (Fig. 6c). As the expression of IL-10 at the lesional sites was diffuse, individual cells could not be counted and hence, a correlation, if any, between PD-1 and IL-10 was not assessed.

## Discussion

CD8^+^ naive T-cells, being mediators of pathogen control, are targets for microbial modification. To achieve this, they orchestrate differentiation via induction of cytotoxic/effector cells that in turn release IFNγ, activate macrophages and dendritic cells, as also promote differentiation of CD4^+^ T-cells^38^. In dermal Leishmaniasis, CD8^+^ T-cells show a diverse disease repertoire with varying intensities of inflammatory infiltrate, and depending on the parasite species, can be protective or deleterious^39^. In patients with localised self healing CL (LCL) caused by *L*. *major*, an increased proportion of CXCR3/CD4^+^ and/or CD8^+^ T-cells provided a protective role^40^, whereas, in severe forms of CL caused by *L*. *braziliensis*, increased disease severity occurred secondary to dysregulated CD8^+^ T-cell cytotoxicity^41^. Studies have shown skin-resident, IFN-γ producing CD4^+^ T-cells protect against *L*. *major*, via recruitment and activation of inflammatory monocytes that mediate parasite elimination by production of reactive oxygen species and nitric oxide^42^. Conversely, in patients with diffuse CL (DCL) caused by *L*. *mexicana*, the uncontrolled spread of the parasite was associated with an enhanced presence of exhausted CD8^+^ T-cells^43^. In a head on comparison of LCL vs. DCL, it was demonstrated that CD8^+^ T-cells sourced from DCL showed a significant reduction in their effector responses *vis-à-vis* their self healing variant^43^. In self healing PKDL in East Africa/Sudan, a preponderance of CD4^+^ T-cells *vis-a-*vis its CD8^+^ variant was demonstrated^44^. In contrast, in Indian non self-healing PKDL, although there was a massive influx of CD3^+^ T-cells^20^, it comprised primarily of CD8^+^, with a near total absence of CD4^+^ T-cells (Fig. 1) that possibly facilitated development of a pro-parasitic milieu and disease sustenance. This absence of CD4^+^ T-cells can be attributed to the enhanced presence of alternatively activated macrophages, that via IL-4/IL-13 mediated activation of Stat-6 inhibit T-cell proliferation and survival^45–47^.

In Leishmaniasis, the inhibition of IL-12 along with an induction of IL-10 and TGFβ underlies the bias towards a Th2 response^11^. In PKDL, the enhanced secretion of IL-10 and TGF-β by antigen-stimulated PBMCs has been associated with disease severity^18^. Cellular infiltration is common to Sudanese and South Asian PKDL (Supplementary Fig. S2), but the absence of granuloma in the latter can be attributed to the IL-10 rich milieu (Fig. 6), which can inhibit granuloma formation as demonstrated in tuberculosis^48^. Furthermore, the resultant microenvironment of IL-4/IL-13 and IL-10 facilitated the emergence of alternatively activated M2 monocytes/macrophages^23^, that in turn supported the expression of Th2 chemoattractants, CCL17 and CCL22 (Fig. 2). These chemokines upon interaction with their receptor CCR4, facilitate migration of skin homing T-cells to the pathologic site, and was endorsed in PKDL by the increased presence of IL-5 and IL-10 (Fig. 6)^37^. Post treatment in a few cases, the levels of CCL17 and CCL22 remained increased or unchanged, that may be attributed to their longer duration of disease (6–12 years) *vis-a-vis* the median disease duration of 2.71 years. The enhanced frequency of CD8^+^CCR4^+^ T-cells in circulation and dermal lesions promoted the dermal extravasation of CD8^+^ T-cells (Fig. 3).

In CL, the higher expression of CXCR3 *vis-a-vis* CCR4 accounted for their self healing propensity^40^. This was corroborated in CL caused by *L*. *braziliensis*, wherein during the early phase of the disease, there is a raised expression of CXCR3^49^. However, in peripheral blood and dermal lesions of PKDL cases, the frequency of CXCR3^+^ remained unaltered as compared to healthy individuals (Supplementary Fig. S4). Subsequently, in the late stage of the disease, a higher expression of CCL17 and CCR4 has been reported, suggestive of a preferential recruitment of regulatory T-cells that facilitated disease progression^49,50^.

A blockade of costimulation can result in the induction of T helper cell anergy and subsequent differentiation of antigen-specific CD8^+^ T suppressor/regulatory cells. Accordingly, it may be envisaged that in PKDL, the enhanced frequency of IL-10 producing antigen-specific circulating CD8^+^ T-cells^15^ and CD8^+^CD28^−^ T-cells^20^, along with impairment of antigen specific proliferative responses^20^ caused attenuation of the effector CD8^+^ T-cell responses in the dermal lesions, as evident by the conspicuous absence of Perforin and/or Granzyme positivity^51^ (Fig. 4) and absence of tissue damage. This was corroborated by absence of the intracellular signaling molecule, Zap-70 which is essential for TCR mediated CD8^+^ T-cell activation and cytolytic activity^31^. Additionally, as the decreased presence of CD127 or the IL7 receptor is associated with an increased rate of disease progression and T-cell exhaustion^52^, its reduced expression (Fig. 4) endorsed the impaired activation status of CD8^+^ T-cells in PKDL.

**Figure 7:**
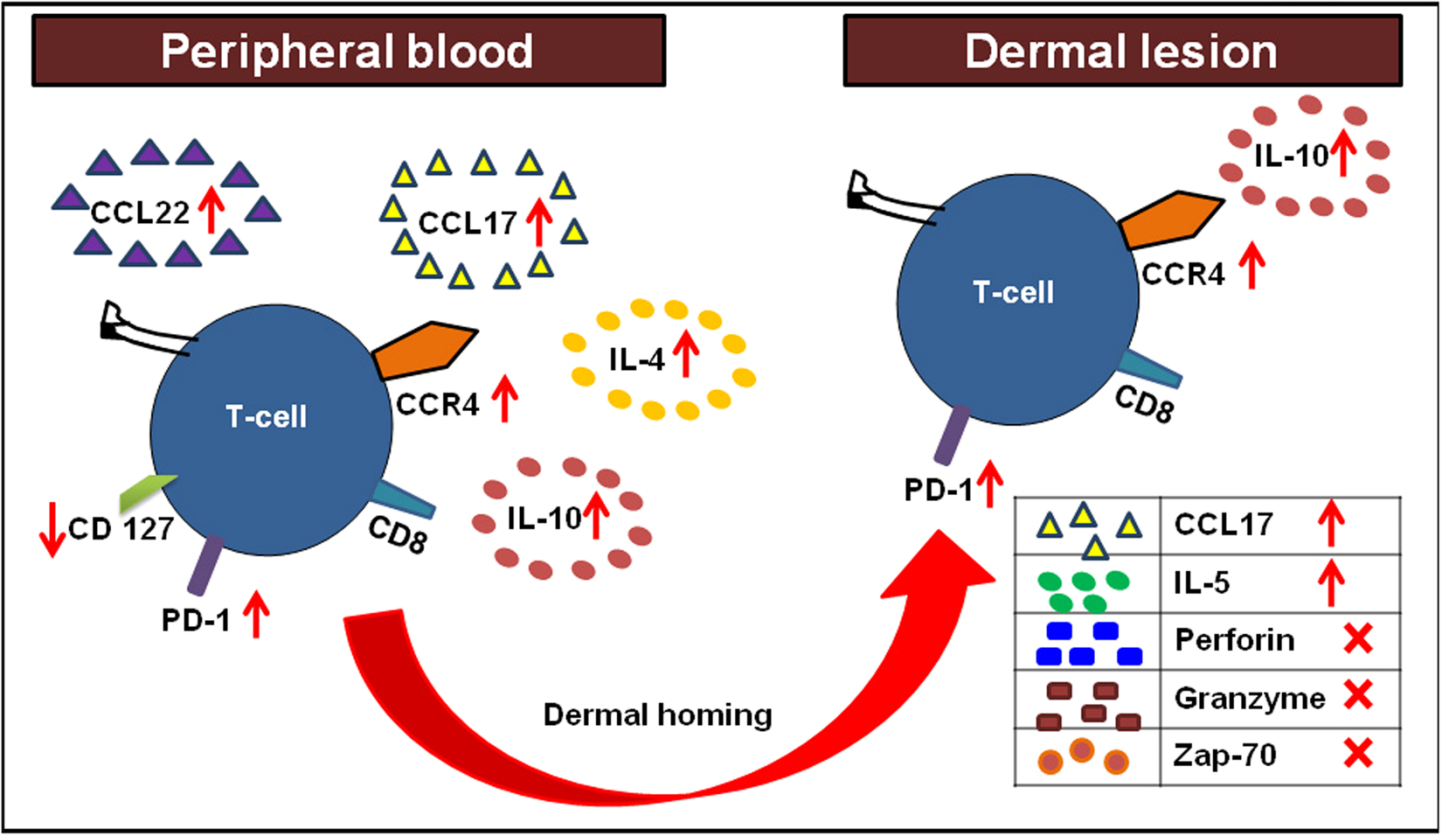
Cellular interactions between peripheral blood and dermal lesions in patients with PKDL. In patients with PKDL, the increased presence of CCL17/22 upon interaction with CCR4 paved the way for homing of CD8^+^ T-cells to the dermal lesional sites. These CD8^+^ T-cells showed exhaustion as confirmed by the increased presence of PD-1 in peripheral blood and dermal lesions, which benefitted from the milieu generated by the increased presence of IL-4/IL-5 and IL-10.

The activation of CD8^+^ and CD4^+^ T-cells is tightly regulated by an inhibitory receptor, Programmed death PD-1^53^. In their efforts to survive and proliferate, the *Leishmania* protein gp63 employs immune-escape markers, such as activation of SHP-1 phosphatases, increased PD-1 functions and collectively suppresses T-cell responses^54^. In an experimental model of VL, Joshi *et al*.^55^ demonstrated that *L*. *donovani* parasites can evade CD8^+^ T-cell responses via induction of functional exhaustion. This was substantiated in human VL wherein splenic CD8^+^ T-cells displayed an anergic phenotype, leading to an immunological imbalance and a breakdown of protective responses^56^. In patients with non self healing DCL, the enhanced expression of PD-1 was associated with a pronounced non-responsiveness of CTLs and disease progression^43^. The scenario was similar in PKDL as CD8^+^ T-cells showed an enhanced presence of PD-1 in peripheral blood and dermal lesions (Fig. 5), thereby creating an environment conducive for parasite survival. Ideally, functional assays would provide confirmatory evidence of the non-responsiveness of these PD-1 expressing CD8^+^ T-cells, but obtaining the requisite number of cells would pose a logistic challenge. Taken together, in PKDL, the increased presence of CCL17 and CCL22 in circulation accounted for the dermal homing of the CD8^+^ CCR4^+^ T-cells to the lesional sites, while the upregulation of PD-1 and IL-10 suggested exhaustion, collectively promoting disease progression (Fig. 7). Thus, *Leishmania donovani* parasites have deviously evolved immune escape mechanisms emphasizing the importance of designing immunotherapeutic strategies aimed at restoring effector responses.

## Materials and Methods

### Reagents

Materials were from Sigma Aldrich (St. Louis, Mo, USA) except rK39 immunochromatographic test strips (InBios International, Seattle, WA, USA), anti-human CD4 (clone 4B12, Novocastra, Newcastle upon Tyne, UK,), Chemokine Receptor 4 (CCR4, clone 1G1), CD3 Peridinin chlorophyll (PerCP, clone SK7), CD8 Alexa Fluor 488 (clone RPA-T8), CD8 PerCP (clone SK1), CCR4 Phycoerythrin (PE, clone 1G1, BD Biosciences, San Jose, CA, USA), PD-1(clone EH12.2H7) and CD127 (clone A019D5) APC (Biolegend, San Diego, CA, USA), IL-5 (clone 9906, R&D Systems, Minneapolis, MN, USA), CCL17 (clone N-20), IL-10 (clone E-10), PD-1 (clone C-20), Perforin H (clone H-315), Granzyme B (clone 2C5, Santa Cruz Biotechnology, Dallas, TX, USA), antibody diluent, Target Retrieval Solution Citrate pH 6 (S2369), secondary detection system EnVision™ G|2 System/AP-Rabbit/Mouse (Permanent Red), EnVision™ FLEX Target Retrieval Solution, EnVision™ DuoFLEX Doublestain System (Dako, Glostrup, Denmark), RNA*Later* and RNAqueous kit (Ambion, Austin, TX, USA), One-step reverse transcription kit from Qiagen (Hilden, Germany) and Bio-Plex Pro™ Human Chemokine Panel 40-Plex (BioRad, Hercules, CA, USA).

### Study population

Patients clinically diagnosed as PKDL (n = 20) were recruited from the Dermatology outpatient department, School of Tropical Medicine, Kolkata, India. The initial diagnosis was based on clinical features, a prior history of VL, rK-39 positivity, or if resident in an area endemic for VL. Diagnosis was confirmed by ITS-1 PCR and/or Giemsa staining in dermal biopsies. Patients were randomly allocated to receive SAG (20 mg/kg bw/day i.m., 4 months, n = 8) or Miltefosine (100 mg/day p.o., 4 months, n = 12). Age and gender-matched healthy volunteers (n = 10) were recruited from non-endemic and endemic areas and were negative for anti-leishmanial antibodies and ITS-1 PCR^57^. A dermal biopsy (4 mm) was obtained using a punch biopsy by anaesthetizing the area with lignocaine and venous heparinised blood (5 ml) was collected at disease presentation and completion of treatment. Due to limited availability of biological material, all markers were not evaluated in every patient, and were randomly selected ensuring that at least 5 individuals were studied per assay.

### Immunohistochemistry

For single staining, formalin-fixed paraffin embedded sections (1 µm), were deparaffinised by xylene and rehydrated using alcohol (100–70%) and distilled water. After heat-induced epitope retrieval at pH 6, the slides were incubated for 1 h with the primary antibody (1:40 for CD4, 1:50 for CD8, CCR4, PD-1, IL-10, IL-5, CCL-17, Granzyme B, Perforin H, Zap-70), washed with Tris buffered saline containing 0.05% Tween-20 (pH 7.4) and incubated with EnVision™ G|2 System/AP-Rabbit/Mouse (Permanent Red) as per the manufacturer’s protocol and counter stained with hematoxylin. Sections from human lymph nodes served as positive controls and for healthy controls, 4 mm skin biopsies were obtained from laboratory volunteers or the foreskin of individuals undergoing voluntary circumcision. For dual staining, sections were deparaffinised and dual staining performed using EnVision™ DuoFLEX Doublestain System^21^. Five fields were manually counted under the light microscope (Axioscope A1, Carl Zeiss, Germany) at 400X magnification and expressed as cells mm^−2^. To minimize bias, two blinded investigators independently evaluated the slides.

### Quantification of circulatory CCL17 and CCL22

Plasma levels of CCL17 and CCL22 were measured (diluted 1:4), using a multiplex detection kit (BioRad, Hercules, CA, USA) as per the manufacturer’s protocol. Data was acquired in a Luminex 200 Labmap system (Luminex, Austin, TX, USA) and analysed using Bio-Plex Manager software version 6.2.

### Isolation of total RNA and Reverse transcriptase-PCR analysis

Skin biopsies were stored in RNA*Later* at −80 °C and total RNA isolated as per the manufacturer’s instructions (Ambion, Life Technologies, USA). Reverse transcriptase-PCR was performed on RNA (50 ng) with the one-step reverse transcriptase-PCR kit (Qiagen, Hilden, Germany) using gene-specific primers (10 µM) for β-actin (F, 5′-CCCAAGGCCAACCGCGAGAAAGAT-3′; R, 5′-GTCCCGGCCAGCCAGGTCCAG-3′), and PD-1 (F, 5′-ATGTAGCCGCCCCACACAGA-3′; R, 5′-CATCCATCTTTTTCAGCCAT-3′). After incubation at 50 °C for 30 min. and PCR activation (95 °C, 15 min.), the amplification comprised 35 cycles of denaturing (94 °C, 30 seconds), annealing (30 seconds), extension (72 °C, 60 seconds), and a final extension (72 °C, 10 min.) in a thermocycler (Applied Biosystems, California, USA). Products were resolved on agarose gels (2%) containing ethidium bromide (0.5 μg/ml) and analyzed (G-BOX gel doc, Syngene, Cambridge, UK) using Gene Tools (Version 4.01.04) software, values being normalized to their respective β-actin.

### Immunophenotyping

Whole blood (100 μl) was surface stained with fluorochrome conjugated antibodies to CD69-PE, CD8-PerCP, CCR4-PE, CXCR3-APC, PD1-APC and CD127-APC along with isotype controls^13^ and acquired on a FACS Calibur (BD Biosciences, San Jose, CA, USA)^13^.

### Intracellular detection of perforin and granzyme

Blood (100 µl) was incubated with anti-human CD8-PerCP (20 min., room temperature) followed by a 10 min. incubation with Lyse-fix solution (BD Biosciences, San Jose, CA, USA). After a wash with phosphate-buffered saline (PBS, 0.02 M)-2% fetal bovine serum (FBS) and permeabilization in Cytofix-Cytoperm buffer (BD Biosciences, San Jose, CA, USA, 100 μl, room temperature, 20 min.), antihuman PE-conjugated Perforin and Granzyme along with respective isotypes were added. Following a 15 min. incubation in perm-wash buffer, cells were washed and acquired on a flow cytometer.

### Flow cytometry

Lymphocytes were gated on their characteristic forward vs. side scatter followed by fluorescence; 5000 lymphocytes were acquired and analyzed using CellQuest Pro software (BD Biosciences, San Jose, CA, USA). Fluorescence was evaluated in terms of frequency (% positivity) and expression (geometric mean fluorescence channel, GMFC) in the FL-1 channel for FITC and Alexa 488, for PE in the FL-2 channel, for PerCP in the FL-3 channel and for APC in the FL-4 channel. The lymphocytes were first gated based on their side and forward scatter, the CD8 population was then identified using CD8-PerCP and the frequency of CD127-APC, CD69-PE, Perforin-PE, Granzyme-PE, CCR4-PE, CXCR3-APC and PD1-APC examined within the CD8^+^ T-cells. Frequency was calculated in the entire CD8^+^ population by dividing the percentages of upper right quadrant by the sum of upper and lower right quadrant. Analysis was done for fluorescence by Cell-Quest pro software (BD Biosciences, San Jose, CA, USA).

### Statistical analysis

Results were expressed as median (Interquartile range, IQR) and data analyzed between groups by Kruskal-Wallis Post Dunn test for non-parametric data, while paired data were analyzed using Student’s t-test (for parametric data). Correlation was done using Pearsons correlation for parametric data using GraphPad Prism software (version 5.0, GraphPad software Inc., La Jolla, CA, USA), p < 0.05 being significant.

### Ethics statement

The study received approval from the Institutional Ethics Committee of School of Tropical Medicine, Kolkata (Order No: 1096 dated: 23.05.2009) and Institute of Post Graduate Medical Education and Research, Kolkata (Ref No. Inst/IEC/1893 dated: 06.08.2005) and all experiments were performed in accordance with relevant guidelines and regulations. Individuals or their legally acceptable representative (if <18 years) gave a written informed consent. Informed consent was also obtained to publish after maintaining patient confidentiality information/images in an online open-access publication.

## Supplementary information

Supplementary information

## Data Availability

All data generated or analysed during this study are included in this article and its Supplementary Information.
