# Author Correction: Impaired activation of lesional CD8^+^ T-cells is associated with enhanced expression of Programmed Death-1 in Indian Post Kala-azar Dermal Leishmaniasis

**DOI:** 10.1038/s41598-019-48640-0

**Published:** 2019-09-25

**Authors:** Shibabrata Mukherjee, Ritika Sengupta, Debanjan Mukhopadhyay, Claudia Braun, Sneha Mitra, Susmita Roy, Nilay Kanti Das, Uttara Chatterjee, Esther von Stebut, Mitali Chatterjee

**Affiliations:** 1 Department of Pharmacology, Institute of Postgraduate Medical Education and Research, Kolkata, 700020 India; 2 Department of Dermatology, University Medical Center, Johannes Gutenberg University, Mainz, 55131 Germany; 3 Department of Dermatology, Calcutta Medical College, Kolkata, 700073 India; 4 Department of Pathology, Institute of Postgraduate Medical Education and Research, Kolkata, 700020 India; 5 Klinik für Dermatologie und Venerologie, Universitätsklinikum Köln, 50937 Koln, Germany; 6 Present Address: Department of Pathology, Microbiology and Immunology, School of Veterinary Medicine, University of California, Davis, USA

Correction to: *Scientific Reports* 10.1038/s41598-018-37144-y, published online 24 January 2019

The original version of this Article contained a typographical error in the writing of the author Esther von Stebut, which was incorrectly given as Esther von Stebut-Borschitz. This has now been corrected in the PDF and HTML versions of the Article, and in the accompanying Supplementary Information file.

