# Supplementary material for: Impaired activation of lesional CD8+ T-cells is associated with enhanced expression of Programmed Death-1 in Indian Post Kala-azar Dermal Leishmaniasis
Source: Sci Rep. 2019 Jan 24;9:762. doi: 10.1038/s41598-018-37144-y (PMC6345993; doi:10.1038/s41598-018-37144-y)
Supplement: Supplementary file 1 — Supplementary information [file 41598_2018_37144_MOESM1_ESM.pdf]

Impaired activation of lesional CD8$^+$ T-cells is associated with enhanced expression of Programmed Death-1 in Indian Post Kala-azar Dermal Leishmaniasis

Shibabrata Mukherjee[1], Ritika Sengupta[1], Debanjan Mukhopadhyay[#1], Claudia Braun[2], Sneha Mitra[1], Susmita Roy[1], Nilay Kanti Das[3], Uttara Chatterjee[4], Esther von Stebut[5] and Mitali Chatterjee[1*]

[1]Dept. of Pharmacology and [4]Pathology, Institute of Postgraduate Medical Education and Research, Kolkata-700020, India

[2]Dept. of Dermatology, University Medical Center, Johannes Gutenberg University, Mainz-55131 Germany

[3]Dept. of Dermatology, Calcutta Medical College, Kolkata-700073, India

[5]Klinik für Dermatologie und Venerologie, Universitätsklinikum Köln, 50937 Koln, Germany

[#]Present address: Department of Pathology, Microbiology, Immunology. School of Veterinary Medicine, University of California, Davis, USA.

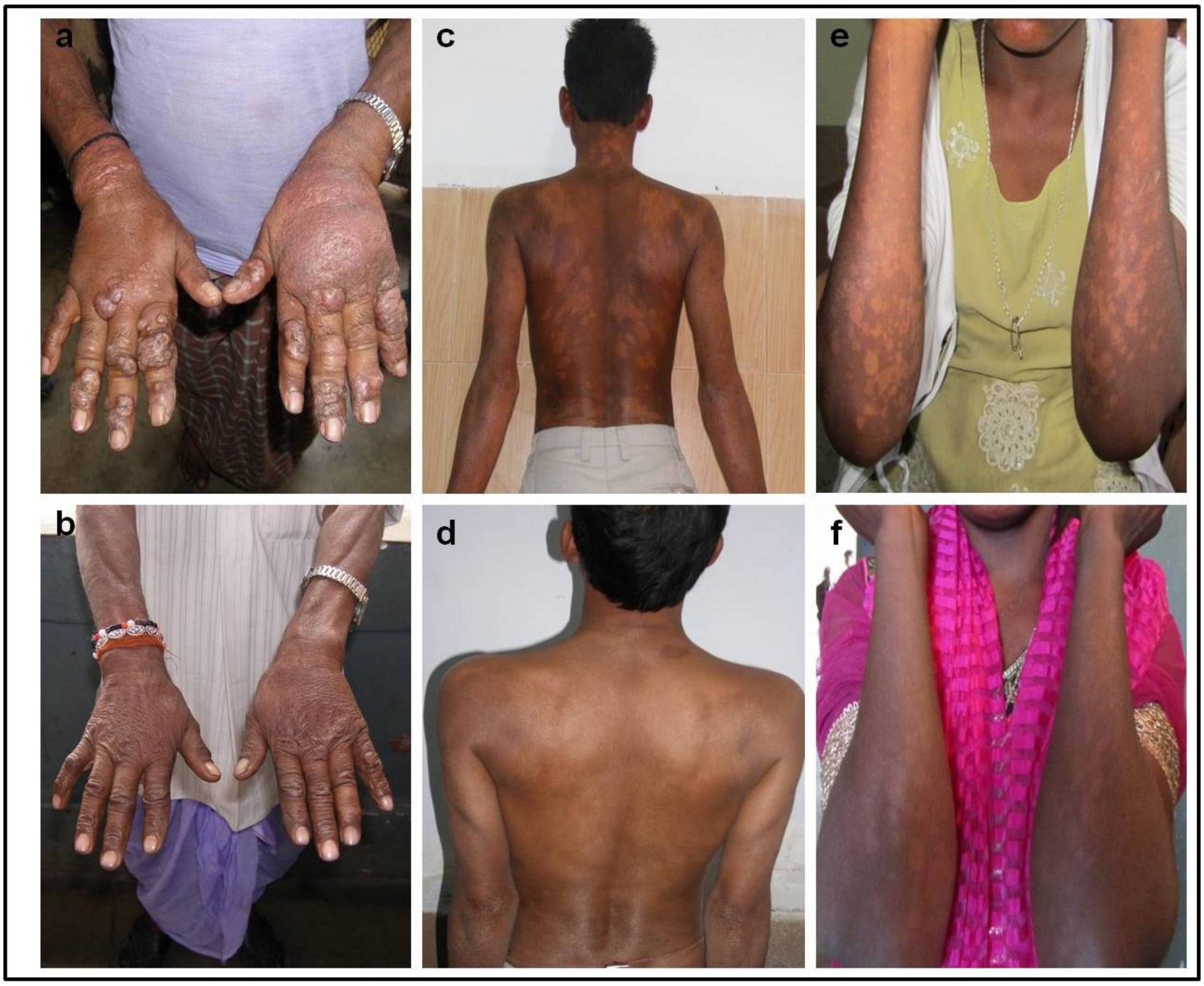

Supplementary Figures S1, a-f: Distribution of lesions in patients with PKDL at disease presentation and post treatment

a & b: Representative profile of a patient with polymorphic PKDL at disease presentation and after completion of treatment

c-f: Representative profiles of two patients with macular PKDL at disease presentation (**c, e**) and after completion of treatment (**d, f**)

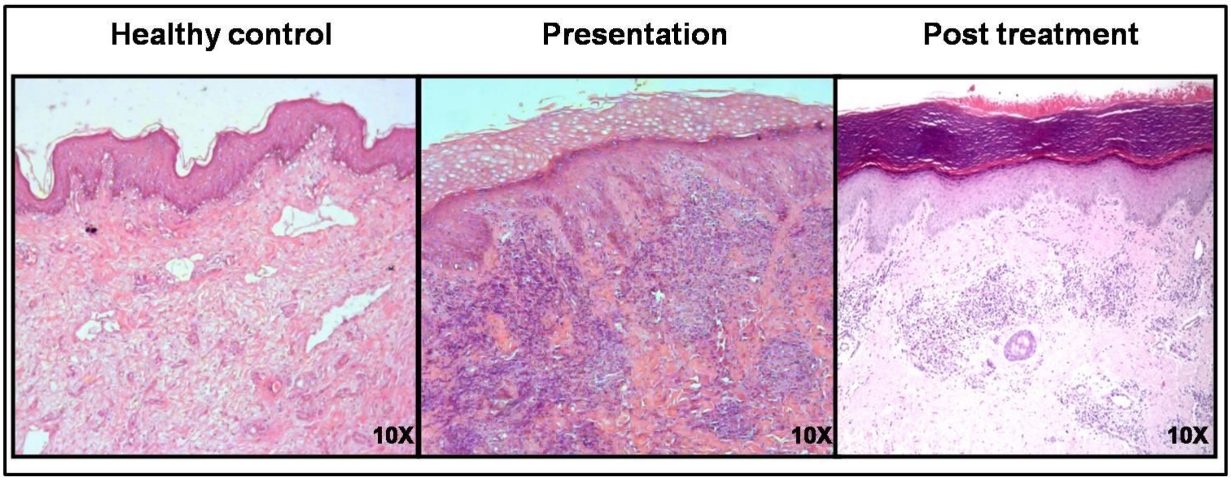

Supplementary Figure S2: Representative profile of H&E-stained sections of a healthy control along with a patient with PKDL at disease presentation and post-treatment (10X magnification)

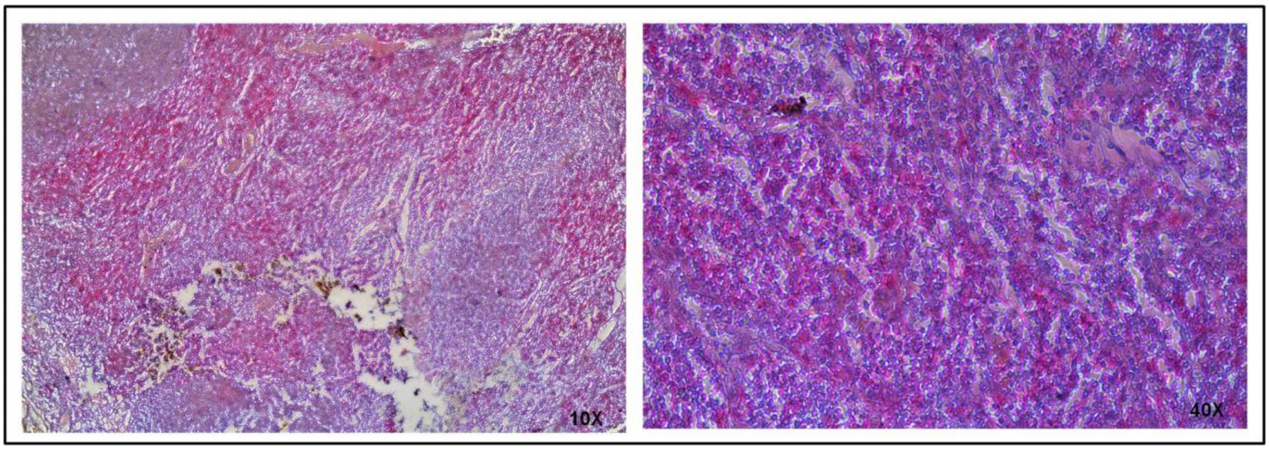

Supplementary Figure S3: Representative profile of human lymph node (positive control) showing presence of $CD4^{+}$ T-cells (10X and 40X magnification)

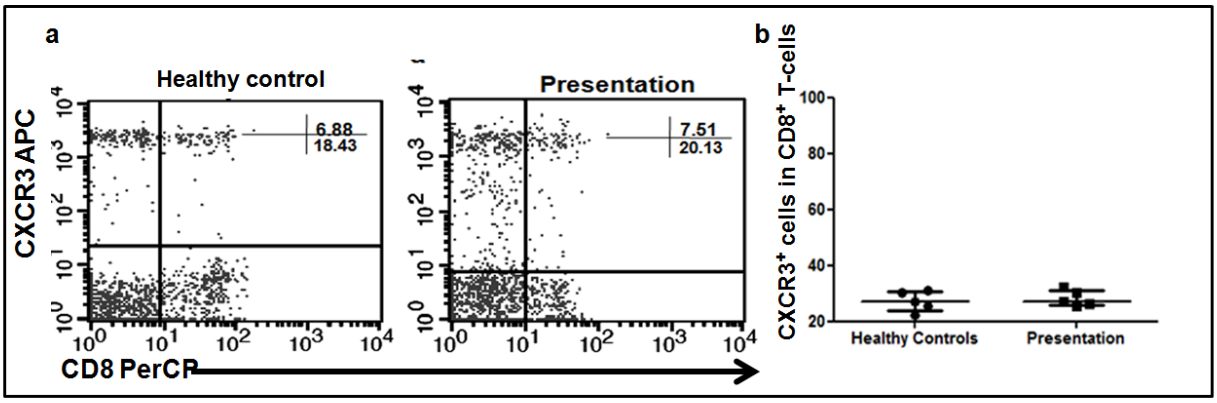

Supplementary Figures S4 a & b: Status of CXCR3$^+$ in CD8$^+$ T-cells in peripheral blood of patients with PKDL

a. Representative quadrant plots showing frequency (%) of CXCR3$^+$ in CD8$^+$ T-cells of a healthy control and patient with PKDL.

b. Scatter plots showing the frequency (%) of CXCR3$^+$ in CD8$^+$ T-cells of healthy controls (●, n=5) and patients with PKDL at presentation (■, n=5). The proportion of CXCR3$^+$ in the entire CD8$^+$ T-cell population was calculated by dividing the percentages of upper right quadrant by the sum of upper and lower right quadrant. Each horizontal bar represents the median value.

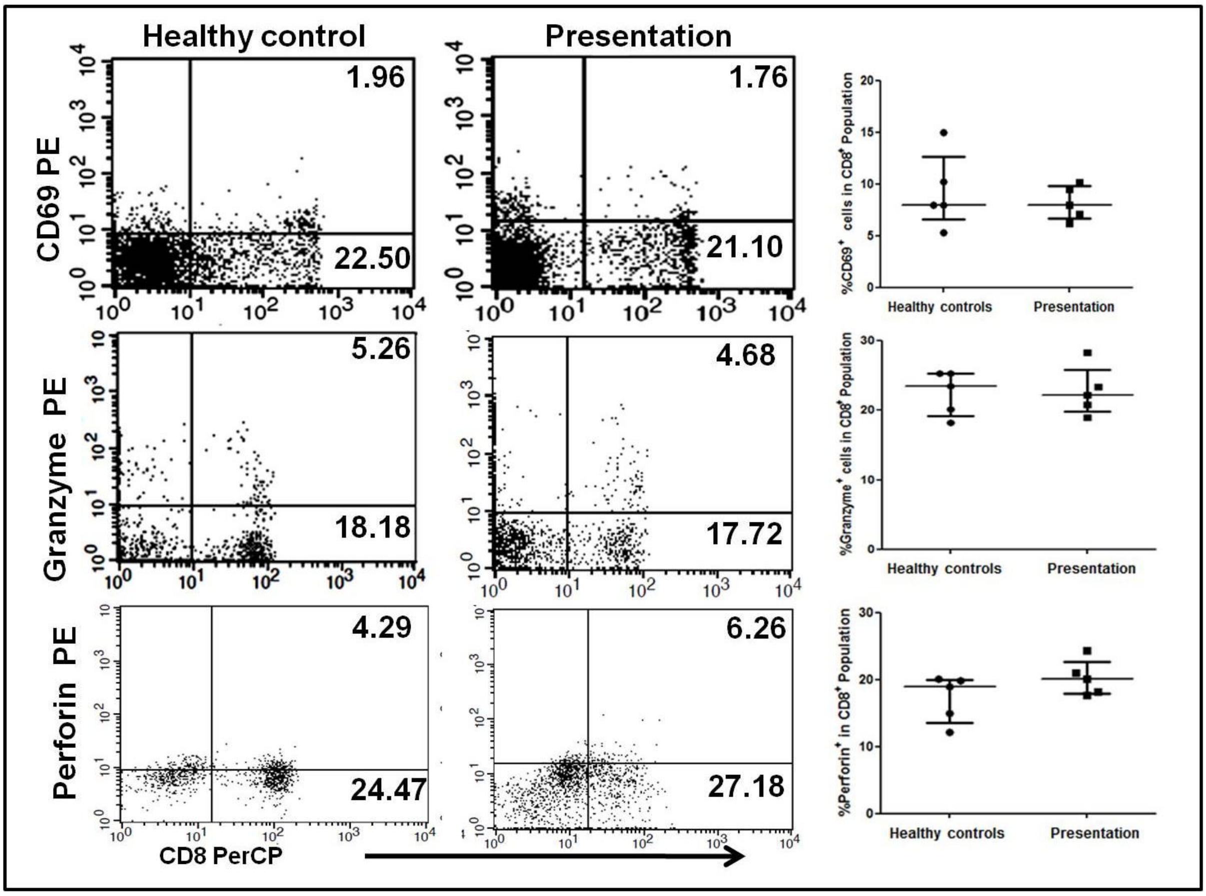

Supplementary Figure S5: Status of CD69, Granzyme and Perforin in $CD8^+$ T-cells in peripheral blood of patients with PKDL

Representative quadrant plots indicating frequency (%) of $CD69^+$, Granzyme and Perforin along with scatter plots within $CD8^+$ T-cells of healthy controls (●, n=5) and patients with PKDL (■, n=5).

The proportion of $CD69^+$, $Granzyme^+$ and $Perforin^+$ in the entire $CD8^+$ T-cell population was calculated by dividing the percentages of upper right quadrant by the sum of upper and lower right quadrant. Each horizontal bar represents the median value.

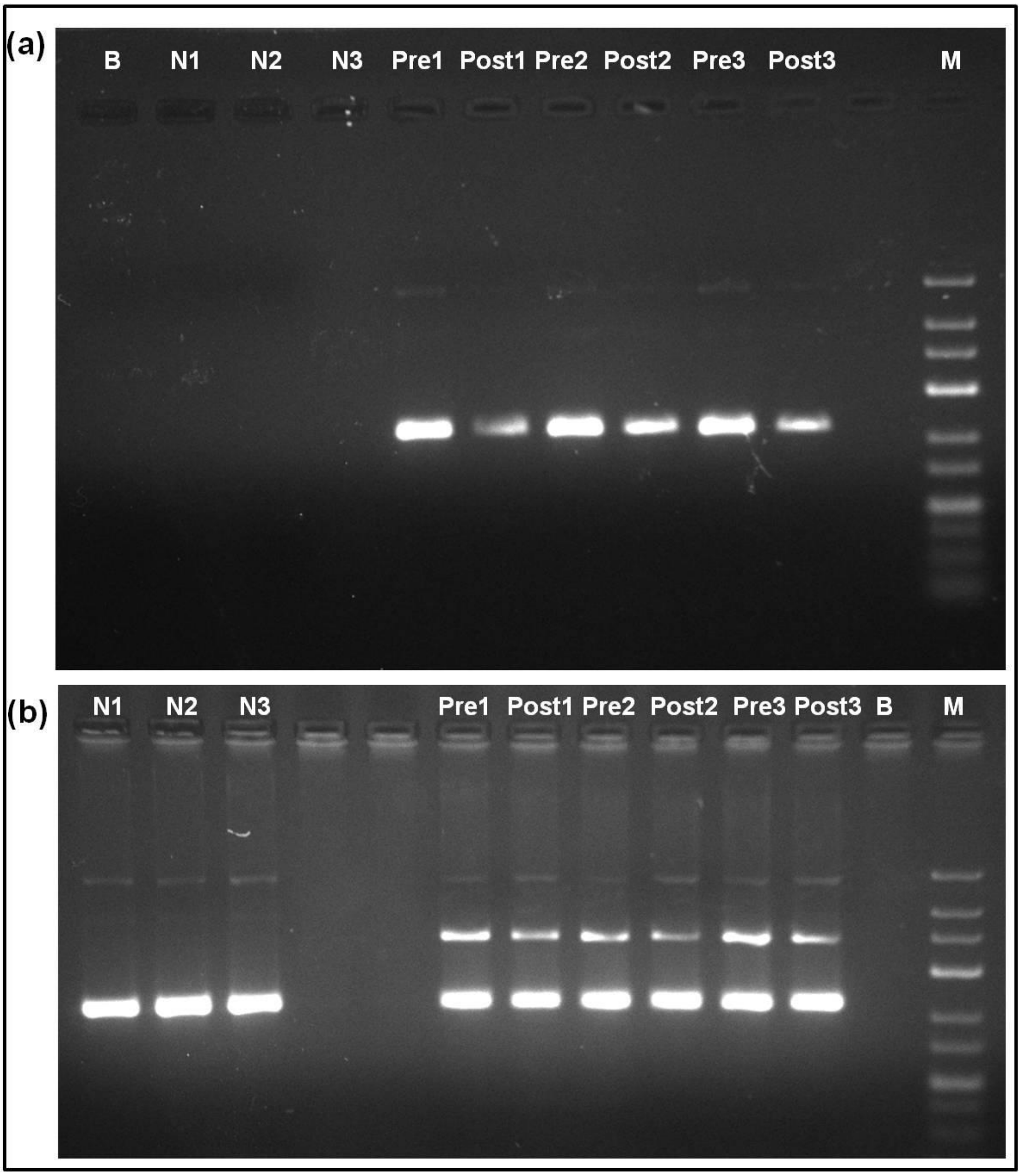

Supplementary Figure S6 a & b: Full length gel images showing mRNA expression of PD-1 in dermal biopsies of patients with PKDL

a. Full-length gel image for mRNA expression profile of PD-1 in dermal biopsies of healthy controls (N1-3), patients with PKDL (Pre1-3) and post treatment (Post1-3); B- Blank, M- Molecular weight marker

b. Full-length gel image for mRNA expression profile of β-actin in dermal biopsies of healthy controls (N1-3), patients with PKDL (Pre1-3) and post treatment (Post1-3); B- Blank, M- Molecular weight marker

Samples were derived from the same experiment and run in two different gels (PD-1 and β-actin) and the gels were processed in parallel under same experimental conditions
